# The interaction of macrophages and CD8 T cells in bronchoalveolar lavage fluid is associated with latent tuberculosis infection

**DOI:** 10.1080/22221751.2023.2239940

**Published:** 2023-08-02

**Authors:** Qianting Yang, Furong Qi, Taosheng Ye, Jinpei Li, Gang Xu, Xiaomeng He, Guofang Deng, Peize Zhang, Mingfeng Liao, Kun Qiao, Zheng Zhang

**Affiliations:** a Institute for Hepatology, National Clinical Research Center for Infectious Disease, Shenzhen Third People’s Hospital; The Second Affiliated Hospital, School of Medicine, Southern University of Science and Technology, Shenzhen, People’s Republic of China; b Shenzhen Clinical Research Center for Tuberculosis, Shenzhen, People’s Republic of China; c Department of Respiratory endoscopy, Shenzhen Third People’s Hospital, Shenzhen, People’s Republic of China; d Department of Pulmonary Medicine & Tuberculosis, Shenzhen Third People’s Hospital, Shenzhen, People’s Republic of China; e Department of Thoracic Surgery, Shenzhen Third People’s Hospital, Shenzhen, People’s Republic of China

**Keywords:** Tuberculosis, Single-cell RNA sequencing, Macrophage, CD8T cell, Immunoregulation

## Abstract

Mycobacterium tuberculosis (Mtb) infection, including active tuberculosis (TB) and latent Mtb infection (LTBI), leads to diverse outcomes owing to different host immune responses. However, the immune mechanisms that govern the progression from LTBI to TB remain poorly defined in humans. Here, we profiled the lung immune cell populations within the bronchoalveolar lavage fluid (BALF) from patients with LTBI or TB using single-cell RNA sequencing (scRNA-seq). We found that Mtb infection substantially changed the immune cell compartments in the BALF, especially for the three subsets of macrophages, monocyte macrophage (MM)-CCL23, MM-FCN1, and MM-SPP1, which were found to be associated with the disease status of TB infection. Notably, MM-CCL23 cells derived from monocytes after stimulation with Mtb were characterized by high levels of chemokine (*CCL23* and *CXCL5*) production and might serve as a marker for Mtb infection. The MM-CCL23 population mainly recruited CD8-CCR6 T cells through CCL20/CCR6, which was a prominent feature associated with protection immunity in LTBI. This study improves our understanding of the lung immune landscape during Mtb infection, which may inform future vaccine design for protective immunity.

## Introduction

Mycobacterium tuberculosis (Mtb) infection accounts for approximately 1.5 million deaths every year, prompting efforts to develop new host-directed therapies for the treatment of tuberculosis (TB) [1]. However, these efforts have been hindered by an incomplete understanding of the mechanism through which the human immune system responds to Mtb infections.

After Mtb infection, most of the infection is removed by the human immune system, but in some cases the infection may lead to latent Mtb infection (LTBI). Most infected people are asymptomatic, however, 5–10% of those with LTBI exhibit active disease during their lifetime [2]. This could be related to the distinct immune resistance in humans. However, the immune mechanisms that govern the progression of LTBI to TB remain poorly defined. Mtb is engulfed by phagocytic cells after inhalation into the lungs where Mtb infection is initiated. Identifying the molecular mechanisms underlying bacterial clearance or persistence in the lungs and other human respiratory tissues is critical. However, it is extremely difficult to obtain lung tissue samples from Mtb-infected populations, particularly those with LTBI. Several previous studies have used animal models, including monkeys and mice, to simulate human immune responses against Mtb infection [3, 4]. However, owing to the differences in the immune responses of certain species, the latent infection state in animal models remains controversial.

In this study, we collected the alveolar lavage fluid (BALF) from LTBI and TB and performed single-cell RNA sequencing (scRNA-seq) to profile the immune cell BALF. We found that the interaction between monocyte macrophage (MM)-CCL23 and CD8-CCR6 was characteristic of LTBI status. The discovery of this lung immune landscape is expected to improve our understanding of immune protection against Mtb infection and indicates that CD8-CCR6 T cells might be useful potential targets for TB control.

## Materials and methods

### Subjects and clinical sample collection

The medical ethics committee of Shenzhen Third People’s Hospital approved the study, and all subjects provided written informed consent. All samples were collected from Shenzhen Third People’s Hospital; the sample details and definitions are provided in Supplementary Table 1.

### Library construction and sequencing

The scRNA-seq libraries were prepared with Chromium Single Cell VDJ Reagent Kits (10x Genomics) following the manufacturer’s instructions. Briefly, gel beads in emulsion (GEM) were generated by combining barcoded Gel Beads, a Master Mix containing 20,000 cells, and Partitioning Oil onto Chromium Chip B. Reverse transcription occurred inside each GEM, after which cDNAs were pooled for amplification and library construction. The resulting library products consisted of Illumina adapters and sample indices, allowing pooling and sequencing of multiple libraries on the next-generation short-read sequencer. The constructed libraries were sequenced using the NoveSeq6000 platform.

### Cytokine measurement by cytometric bead array (CBA)

Twelve cytokines were detected according to the manufacturer’s instructions (Uni-medica). The data were obtained using flow cytometry (Canto II, BD) and analyzed using LEGENDplex v8.0 (VigeneTech Inc.).

### Cell culture for human blood-derived macrophages and stimulation

CD14-positive monocytes were isolated from peripheral blood mononuclear cells (PBMCs) using human CD14 microbeads (Miltenyi Biotec) and differentiated to macrophages for 7 d with M-CSF (10 ng/mL; PeproTech). H37Rv (MOI = 5), IL-6, TNF-α, and/or IFNγ (20 ng/mL; PeproTech) were then added, and the solution was incubated for another 24 h. The cells were then collected for bulk-seq.

### Real-time PCR

Total RNA was extracted from human PBMCs (Qiagen) following the manufacturer’s instructions. Purified RNA was reverse transcribed to cDNA using the PrimeScript RT reagent kit (TaKaRa). Quantitative PCR (qPCR) was performed using SYBR Green PCR Master Mix (TaKaRa) following a standard protocol. The relative mRNA expression of the target genes was calculated by comparing it with the expression of the glyceraldehyde-3-phosphate dehydrogenase (GAPDH) housekeeping gene using the 2-ΔΔCt method. The primers were as follows: human GADPH sense, 5′’-AGATGGCACGGGACACTACCTG-3′; human GADPH antisense, 5′-TCGCTTGGGCTTAATGAGGG-3′; human CXCL-9 sense, 5′-TGAGAAAGGGTCGCTGTTCC-3′; human CXCL-9 antisense, 5′-GGGCTTGGGGCAAATTGTTT-3′. human CCL-23 sense, 5′-TCTCATGCTGCAGGATTCCAT-3′; human CCL-23 antisense, 5′-GGTGAGGAAGATGACACCCG-3′.

### Bulk RNA-seq analysis

Raw sequenced reads were mapped against the human genome (GRCh38) using the STAR software with default settings. The gene expression levels (TPM value) of each sample were calculated using the RSEM software. DESeq2 was used to identify the genes differentially expressed between cytokine/bacteria-stimulated and mock-stimulated cells.

### Single cell RNA-seq data processing, cell clustering, and dimension reduction

We aligned the sequenced reads against the GRCh38 human reference genome using Cell Ranger (version 6.1.1, 10x Genomics). Cell quality was assessed using the following criteria: (1) the number of sequenced genes is between 200 and 6,000; (2) the total number of UMI per cell is greater than 1,000; and (3) the percentage of mitochondrial RNA is less than 15% per cell.

Data integration, cell clustering, and dimension reduction were performed using Seurat (version 4.0.4). First, we identified 2,000 highly variable genes (HVGs), which were used for subsequent analysis using the FindVariableFeatures function. Next, we integrated different samples using the IntegrateData function, which eliminates technical or batch effects using canonical correlation analysis (CCA). Using these HVGs, we constructed a Principal Component Analysis (PCA) matrix with the top 50 components, using the RunPCA function. The cells were then clustered by the FindClusters function after building the nearest neighbour graph using the FindNeighbors function. Cluster-specific marker genes were identified using the FindMarkers function of the MAST algorithm. The clustered cells were then projected into a two-dimensional space for visualization using the nonlinear dimensional reduction method RunUMAP in the Seurat package.

### Integrated analysis of myeloid, NK, and T cells from the BALF

Myeloid, NK, and T cells from the BALF were integrated separately. For myeloid cells, we extracted macrophage cells from the BALF and monocytes and myeloid dendritic cells (mDC) from PBMCs from the corresponding raw count matrix. The extracted cells were integrated using CCA in Seurat, as described above. For clustering, the resolution parameter was set to 0.8. Similarly, we extracted NK and T cells from the BALF and PBMCs from the corresponding raw count matrix. The extracted cells were integrated using CCA in Seurat, as described above, and the resolution parameter was set to 0.8 for clustering.

### Analysis of differentially expressed genes (DEGs)

The FindMarkers function in Seurat, with the MAST algorithm, was used to analyze the DEGs. For each pairwise comparison, we ran the FindMarkers function with parameters of test.use =  “MAST”. Genes were defined as significantly upregulated if the average logarithm two-fold change (log2FC) was > 0.58 and adjusted *P* < 0.05. Genes with log2FC < −0.58 and adjusted *P* < 0.05 were considered significantly downregulated. ClusterProfiler in programme R was used to perform gene ontology (GO) term enrichment analysis for significantly upregulated and downregulated genes. Only the GO term Biological Process (BP) was displayed.

### Pseudotime trajectory analysis

Trajectories analysis was performed using scVelo for monocyte-macrophages with n_pcs = 30 and n_neighbors = 30.

### Cell–cell interaction analysis

**“**Cell–cell interaction” analysis between cell populations was performed using the R package CellChat [5]. In this process, CellChatDB was set as “Secreted Signaling.”Specifically, we first identified over-expressed ligands or receptors in one cell group using the“dentifyOverExpressedGenes”function. The significant cell–cell communication between cell populations was inferred by the “computeCommunProb” and “computeCommunProbPathway” functions on a signaling pathway level. The dominant senders, receivers, mediators, and influencers in the intercellular communication network were calculated using the “netAnalysis_computeCentrality” function.

### Whole-blood transcriptomic analysis

The MetaIntegrator was used to perform the meta-analysis. Gene expression matrices were prepared for each dataset to determine the effect sizes for the genes. Summary effect sizes were calculated to assess differences in gene expression across distinct clinical groups.

### Data availability

The raw data have been deposited in the Genome Sequence Archive in the National Genomics Data Center, Beijing Institute of Genomics, Chinese Academy of Sciences, under accession number HRA000297 and are publicly accessible at http://bigd.big.ac.cn/gsa-human.

## Results

### Landscape of immune cells in BALF during TB disease progression at the single-cell level

**Figure 1.**
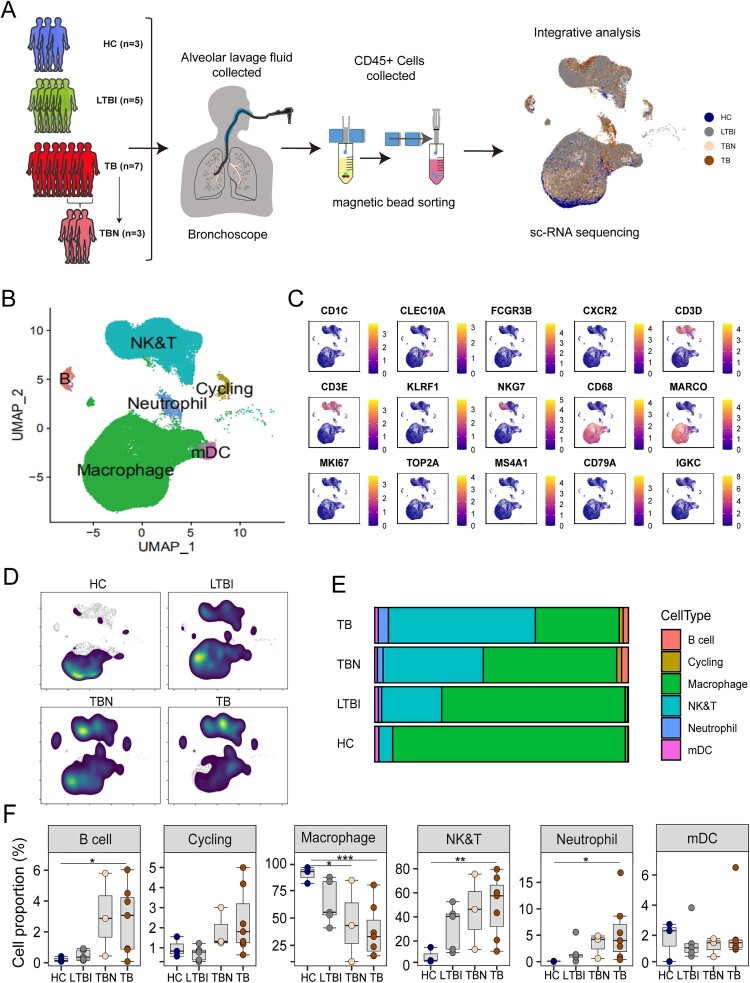
Single-cell transcriptional profiling of BALF cells. (A) Flowchart depicting the overall experimental design of this study. BALF cells were collected from healthy controls (HC, *n* = 3), patients with latent tuberculosis infection (LTBI, *n* = 5), TB in lesion local (TB, *n* = 7), and TB in normal local (TBN, *n* = 3). CD45-positive cells were enriched in the BALF using magnetic bead sorting, and then high-quality single cells were generated to characterize the BALF-derived immune cell landscape. (B) The UMAP projection of the combined BALF cells scRNA-seq dataset identified six major cell types. (C) The expression of representative cell type discriminating genes is indicated. (D) Density plots show the UMAP projection of BALF cells from different groups. (E) The bar plot shows the proportions of each cell type in BALF cells from individual subjects. (F) Box plots showing the proportion of each cell type in BALF cells between individual subjects. Groups are shown in different colours. All box plots display the median, 25th, and 75th percentiles, and whiskers extend to the maximum and minimum data points. Data were analyzed using a two-sided Student’s t-test (*p < 0.05, **p < 0.01, ***p < 0.001).

We collected the BALF from patients with LTBI and TB and assessed cell composition and architecture using scRNA-seq. Data from three healthy control donors (HC) were downloaded from our public database [4]*.* The BALF from seven patients with TB was collected from the diseased regions of the lung and the lesion-offside normal lung tissues from three patients with TB (TBN). To acquire immune cells, CD45-positive cells were enriched in BALF using magnetic bead sorting. In total, 83,084 high-quality single cells were generated to characterize the BALF-derived immune cell landscape (Figure 1(a), Figure S1A).

The clustering analysis revealed six cell types annotated by canonical marker genes (Figure 1(b)), including T and NK cells (*CD3E, CD3D, KLRF1,* and *NKG7*), B cells (*IGKC* and *MS4A1*), neutrophils (*FEGR3A*), myeloid dendritic cells (mDC) (*CD1C* and *CLEC10A*), macrophage/myeloid cells (*CD68* and *MARCO*), and cycling cells (*MKI67*) (Figure 1(b and c), Figure S1B). We found that the most prominent changes in TB samples included an increase in the number of NK and T cells, B cells, neutrophils, and cycling T cells but a reduction in macrophage populations (Figure 1(d–f)). These data showed that Mtb infection substantially changed the immune cell compartments in the BALF.

### ScRNA-seq defines unique macrophage subsets in BALF after Mtb infection

Macrophages are involved in the natural and trained innate immunity against TB. We comprehensively analyzed the heterogeneity of macrophage populations at a single-cell resolution.

**Figure 2.**
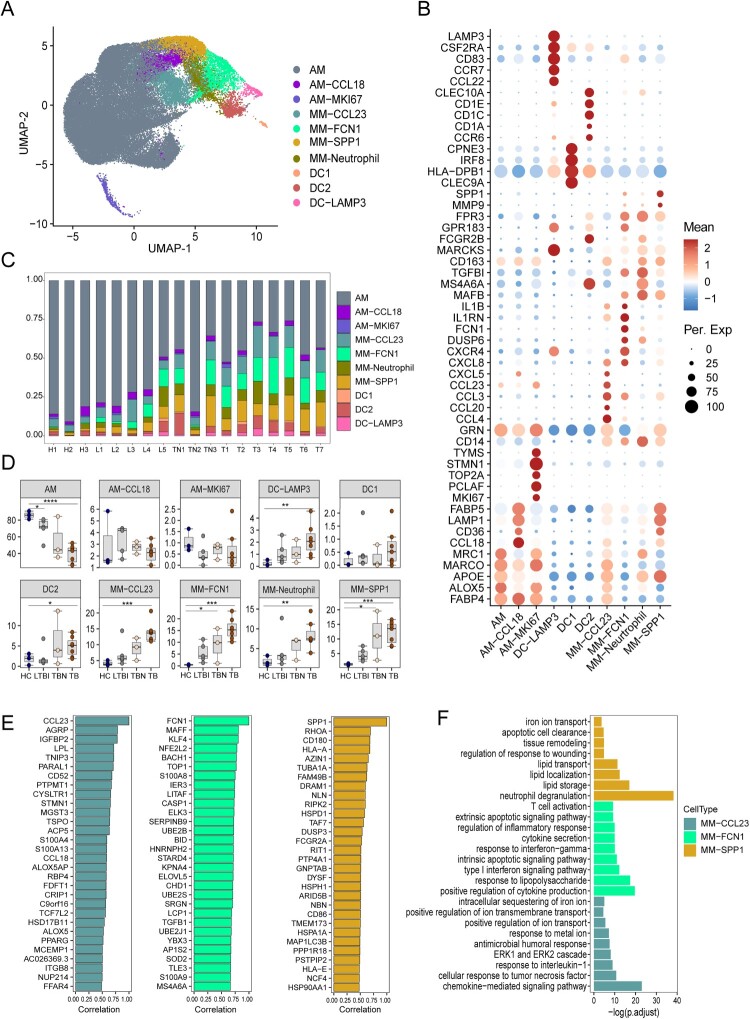
Single-cell analyses of macrophage cell clusters in BALF cells. (A) UMAP plot of the 10 types of macrophages in BALF cells from HC (*n* = 3), LTBI (*n* = 5), TB (*n* = 7) and TBN (*n* = 3). (B) Specific markers for identifying each immune cell type. (C) Bar plot shows the proportions of each cell types in BALF cells from individual subjects. (D) Box plots showing the proportion of each macrophage cell type in BALF cells between individual subjects. Data were analyzed using a two-sided Student’s t-test (*p < 0.05, **p < 0.01, ***p < 0.001). (E) The top 30 differentially expressed genes (DEGs) in different subsets of monocyte macrophage (MM)-CCL23, MM-FCN1, and MM-SPP1 (F) Gene ontology (GO) enrichment analysis by DEGs identified in MM-CCL23, MM-FCN1, and MM-SPP1.

We identified 54,898 myeloid cells (macrophages and mDCs) under all conditions (Figure 2(a), Figure S2A-B). There are two major macrophage subsets, alveolar macrophages (AM) and monocyte-derived macrophages (MM). AM preferentially expressed *ALOX5, APOE,* and *MARCO*. There were two small subsets of AM: AM-CCL18 (*CCL18* and *FABP5)* and AM-MKI67 (*MKI67* and *TOPA2*). The expression of CD14 was observed in MM populations, which were classified as MM-FCN1, MM-SPP1, and MM-CCL23 macrophages annotated by *FCN1*, *SPP1* and *CCL23*, respectively. We also found one MM-neutrophil subset that expressed both MM (*CD14*, *APOE, MARCO)* and neutrophil *(MARCKS, FPR3*, and *FCGR2B)* genes*.* In addition, three DC subsets, DC-LAMP3 (*LAMP3, CSF2RA,* and *CD83*), DC2 (*CD1C, CD1E,* and *CD1A*), and DC1 (*CLEC9A, CPNE3,* and *HLA-DRB1)* were identified (Figure 2(b)). We further confirmed the major functions of these cell clusters using functional signature scores (Figure S2C). AM expressed higher signals related to lipid metabolism (fatty acid oxidation and fatty acid synthesis). DCs expressed genes associated with antigen presentation (MHC-II). MM cells expressed high levels of genes related to cell chemotaxis, inflammation, and IFNs (Figure S2C). In general, the definition of macrophage subsets was reasonable.

We then compared the cell composition of the macrophage subsets across different groups. AM cells showed a significant decrease in both LTBI and TB compared with that in the HC. TB showed the greatest reduction in AM cells. The proportion of DC (DC-LAMP3 and DC2) and MM (MM-CCL23, MM-FCN1, MM-SPP1, and MM-neutrophil) subpopulations was higher in TBN and TB than in HC (Figure 2(c and d)). These data indicate that macrophages exhibit a variety of immune cell compositions and show several changes at different disease stages of Mtb infection.

The most significant changes in macrophages were in MM-CCL23, MM-FCN1, and MM-SPP1, which were largely increased in TB. Gene expression profiles of the three macrophage subtypes were markedly different. MM-CCL23 preferentially expressed a unique gene set, including *CCL23, AGRP*, and *IGFBP2* (Figure 2(e)). We performed gene ontology (GO) analysis using the differentially expressed genes (DEGs) showing upregulated expression in these three MM cells. The chemokine-mediated signaling pathway was the most prominent feature in MM-CCL23 and a series of chemokines (e.g. *CCL23, CCL4,* and *CCL20)* exhibited upregulated expression in MM-CCL23 (Figure 2(f), Figure S2D; Table S2). The upregulated DEGs that exhibited upregulated expression in MM-FCN1 were mainly enriched in the positive regulation of cytokine production, response to lipopolysaccharides, IFN-I, and IFNγ response pathways. TLR genes (e.g. *TLR2*), response to lipopolysaccharide genes (e.g. *CLEC5A*), and cytokine/IFN-stimulating genes (e.g. *IFIT3* and *ISG20*) exhibited upregulated expression in MM-FCN1 cells (Figure 2(f), Figure S2D; Table S2). For MM-SPP1, the pathways of neutrophil deregulation, lipid metabolism, and tissue remodeling showed upregulated expression (Figure 2, Figure S2D; Table S2). Collectively, these three macrophage subsets showed unique immune characteristics. MM-FCN1 exhibited an inflammatory phenotype, MM-SPP1 a tissue repair phenotype, and MM-CCL23 a cell chemotaxis phenotype.

### Immune characteristics of monocyte macrophages in different disease stages of tuberculosis

The increased proportion of these three MM subsets in TB indicates that their function may be associated with Mtb infection stages. We further compared the immune features of these three MM populations between LTBI, TBN, TB and HC.

**Figure 3.**
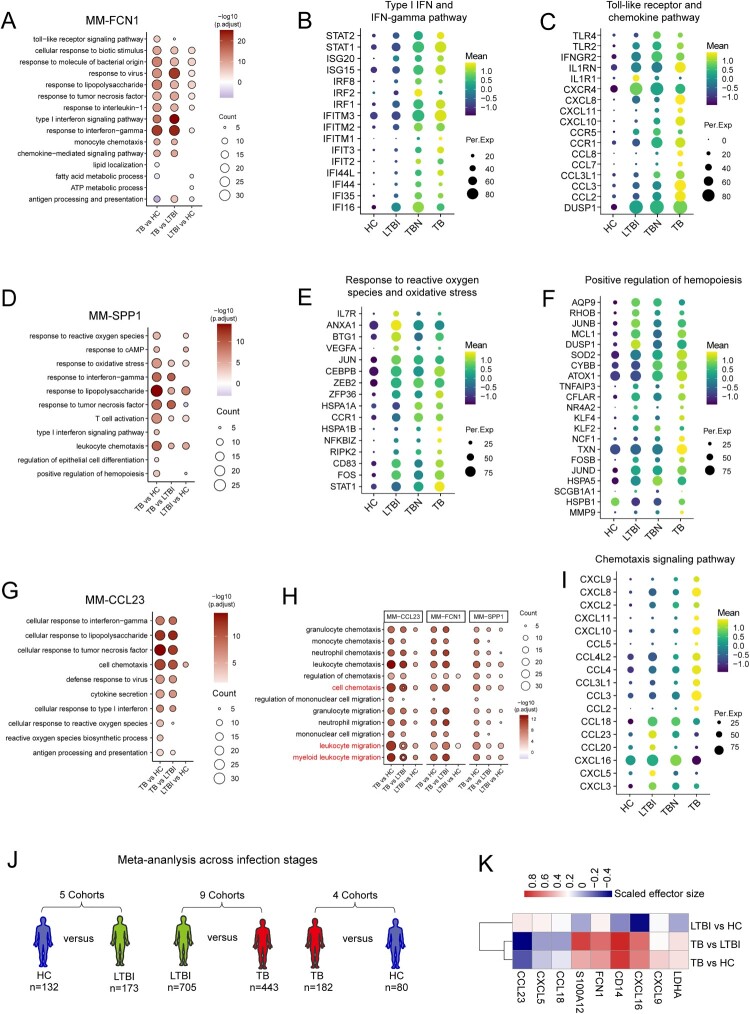
Chemotaxis features of macrophages reflected different states after tuberculous bacterium infection. (A) Enrichment of GO biological process (BP) terms in MM-FCN1 macrophage comparisons between TB and HC, TB and LTBI, and LTBI and HC (selected terms are shown; adjusted *p*-value is indicated by the coloured bar). (B-C) The dot plot of type I interferon and interferon-gamma (B) and toll-like receptor (C) signaling pathway genes of MM-FCN1 macrophages in HC, LTBI, TB, and TBN. (D) BP terms in MM-SPP1 macrophage comparisons between TB and HC, TB and LTBI, and LTBI and HC. (E-F) The dot plot of response to reactive oxygen species and response to oxidative stress (E) and positive regulation of hemopoiesis genes (F) of MM-SPP1 macrophages among different individuals. (G) BP terms in MM-CCL23 macrophage comparisons between TB and HC, TB and LTBI, and LTBI and HC. (H) BP terms of chemotaxis in different subsets of macrophage comparisons are indicated. (I) The dot plot of chemotaxis signaling pathway genes of MM-CCL23 macrophages between different individuals. (J) Conceptual overview of gene expression analysis across clinical infection stages. LTBI (*n*  = 173) versus HC (*n*  = 132), LTBI (*n* = 705) versus TB (*n*  = 443), and TB (*n*  = 182) versus end of HC (*n*  = 80). (K) Heatmap of gene expression (mean effect size) values in different groups. The clinical stage is displayed in rows, and genes are hierarchically clustered and displayed across columns.

The upregulated DEGs in MM-FCN1 were enriched in the toll-like receptor, response to lipopolysaccharide, response to TNF, response to interleukin-1, chemotaxis, IFN-I signaling pathway, and response to IFNγ signaling pathways (Figure 3(a); Table S3). Notably, we found that most of the genes in these pathways were expressed at higher levels in TBN and TB than in LTBI and HC (Figure 3(b and c), Figure S3B-F; Table S3). For example, the expression of genes involved in the IFN-I and IFNγ signaling pathways (e.g. *ISG20* and *IFITM1-3),* TLR signaling pathway (e.g. *TLR4* and *TLR2)* and response to TNF (e.g. *TNFSF13B* and *TNFRSF14*) was the highest in TBN and TB (Figure 3(b and c)). The expression of genes involved in the chemokine response pathway (e.g. *CXCL8* and *CXCL10*) was highest in TB (Figure 3(c)). In contrast, the genes associated with antigen processing and presentation, including *CD74,* and *HLA-DRB5*, were expressed at the lowest levels in TB (Figure S3G). These transcriptional changes implied that MM-FCN1 from TB tended to exhibit phenotypes associated with bacterial recognition and inflammation but showed low levels of antigen presentation activity.

For MM-SPP1, the pathways of the response to lipopolysaccharide, TNF, IFNγ, reactive oxygen species (ROS), oxidative stress, leukocyte chemotaxis, and positive regulation of hematopoiesis were upregulated in TB (Figure 3(d), Figure S3H; Table S3). Notably, *IL7R* (CD127)*, ANXA1,* and *BTG1* were upregulated in LTBI. A previous study found that CD127^high^ monocytes/macrophages retained hypoinflammatory phenotypes within highly inflamed tissue environments [6]. The genes (*STAT1, NFKBIA,* and *HSPA1B)* in the pathways of response to reactive oxygen species and response to oxidative stress were expressed at higher levels in the TB group than in the other groups (Figure 3(e)), whereas the genes (*MMP9, TXN,* and *NCF1*) associated with positive regulation of hematopoiesis were expressed at the lowest levels in TB (Figure 3(f)).

For MM-CCL23, the pathways of cellular response to lipopolysaccharide, TNF, cell chemotaxis, IFN-I, and IFNγ were upregulated in TB when compared to those in LTBI and HC (Figure 3(g), Figure S3I; Table S3). Notably, only cell chemotaxis was evident for MM-CCL23 cells in LTBI compared to that in HC. We comprehensively analyzed the chemotaxis-related pathways in the three MM cell subsets in the different groups. Of note, some of these chemotaxis-related pathways were especially enhanced only in MM-CCL23 cells in LTBI when compared to those in HC (Figure 3(h), Figure S3J). In MM-CCL23 cells, chemokine profiles featured disease status, whereby *CCL20, CCL23, CCL4,* and *CXCL5* were highly expressed in LTBI, and *CXCL8, CXCL9, CCL11*, and *CXCL10* were highly expressed in TB (Figure 3(i)). These chemokine profiles in MM-CCL23 cells were significantly different from those in MM-FCN1. In MM-FCN1, the levels of most chemokines, including *CXCL8*, *CXCL10*, *CXLC11*, *CCL3,* and *CCL2*, significantly increased in TB (Figure 3(c)). The presence of chemokine features observed in MM-CCL23 in the BALF dictates important implications for understanding the protective immunological features of disease progression.

### Distinct chemokine profiles in MM subsets defined LTBI and TB status

Next, we validated whether these chemokine features identified in the BALF could be identified in the blood and correlate these immune features with disease progression. We performed a multi-cohort meta-analysis using MetaIntegrator. In total, 1,549 subjects including HC, LTBI, and TB, across 18 cohorts of publicly available peripheral blood transcriptome profiles, were obtained (Figure 3(j); Table S4). Consistent with our scRNA-seq analysis results, we found that the expression of *CCL23, CCL18,* and *CXCL5* was specifically increased in LTBI compared to that in TB and HC. The expression of *CXCL9, SA10012,* and *CXCL16* was exclusively increased in TB compared to that in LTBI and HC (Figure 3(k)). Furthermore, the expression of *CCL23* and *CXCL9* in the blood samples from different populations was detected using real-time-PCR. Consistent with the transcriptome profiles of the blood, we found that *CCL23* expression was increased in LTBI compared to that in HC the expression of *CXCL9* was increased in TB (Figure S3K). These results support the notion that the changes in chemokine expression in macrophages define a unique stage of TB infection.

### Phenotype of MM in the BALF is similar to that in tuberculosis granuloma

**Figure 4.**
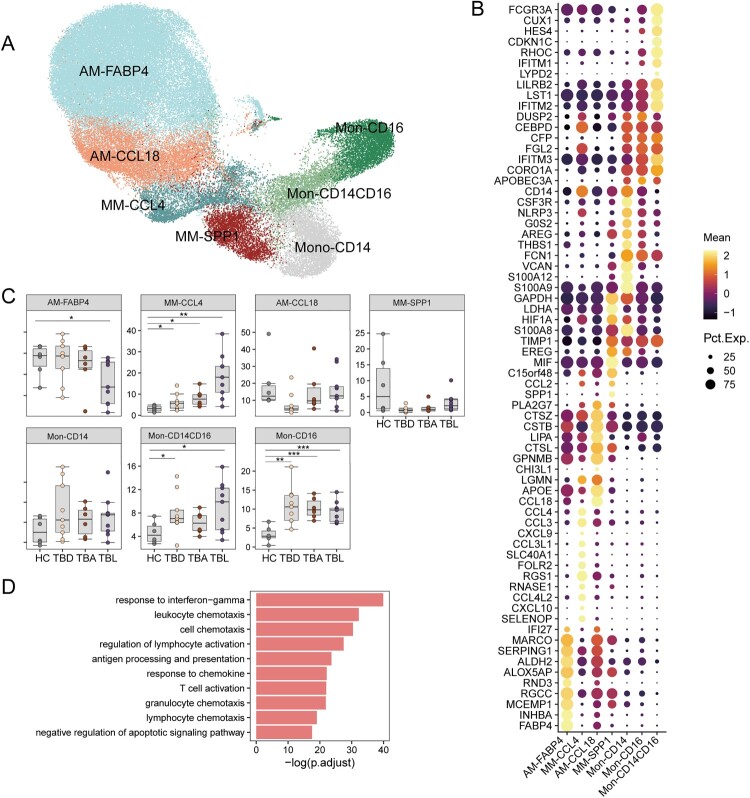
Single-cell analysis of macrophages in tissue cells from HC and TB. (A) UMAP plot of the seven types of macrophages in lung tissues from HC (HC, *n* = 6) and patients with TB. In each patient with TB, tissues that were collected from active lesion tissues were defined as TBL (*n* = 9), tissues from the lesion boundary or adjacent to the active lesion were defined as TBA (*n* = 6), and tissues distal to active lesions (about 2 cm) were defined as TBD (*n* = 9). (B) The specific markers for identifying each immune cell type are indicated. (C) Box plots showing the proportion of each macrophage cell type in tissue between individual subjects. Data were analyzed using a two-sided Student’s t-test (*p < 0.05, **p < 0.01, ***p < 0.001). (D) Differential pathway enriched in different stimulated conditions by GSVA. Top 10 enriched gene ontology terms (Biological Process) in distinct comparisons.

To confirm whether the macrophage populations in the BALF can represent the real state in tuberculosis granuloma, we analyzed the macrophage features of nine TB samples with tuberculosis granuloma (BioProject No. PRJCA013323). In each patient with TB, tissues from the active lesion of TB patients were defined as TBL, tissues from active lesion boundary or adjacent to the active lesion were defined as TBA, and tissues from distal to the active (about 2 cm) were defined as TBD. We identified three macrophage subsets, including AM, MM, and monocyte subsets (monocyte-CD14, monocyte-CD14-CD16, and monocyte-CD16). Two MM subsets, MM-SPP1 and MM-CCL4, were observed and were grouped according to *SPP1* and *CCL4* expression patterns, respectively (Figure 4(a and b)).

When comparing the macrophage features from lung tissue and BALF cells in TB, we found that the gene expression profile of MM-CCL4 in lung tissue was similar to that of MM-CCL23 in BALF, with high expression of a series of chemokine genes, including *CXCL9, CXCL10, CCL4,* and *CCL3*. The expression of MM-CCL4 was higher in TBL than in TBA, TBD and HC (Figure 4(c)). Notably, the DEGs with upregulated expression in MM-CCL4 were enriched in the response to IFNγ and a series of chemokine signaling pathways including leukocyte chemotaxis, cell chemotaxis, response to chemotaxis, and granulocyte and lymphocyte chemotaxis (Figure 4(d)). These results implied that the features of MM-CCL23 in BALF cells were reproducible in lung tissues at different stages of disease.

### Mtb infection induces macrophage differentiation from peripheral monocytes

**Figure 5.**
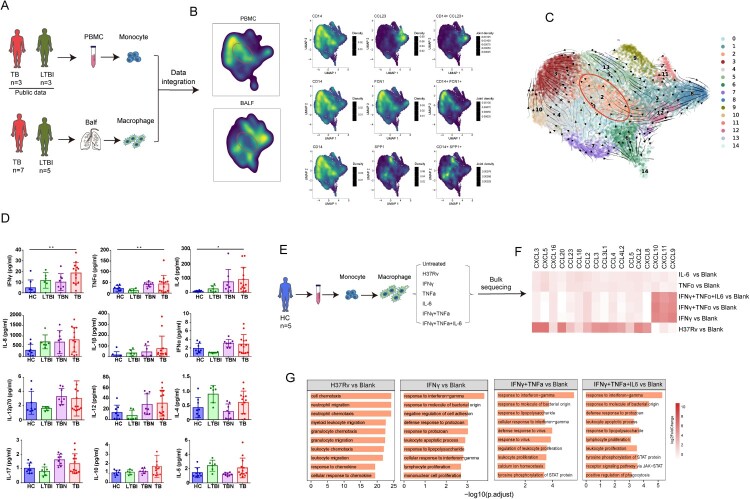
The differentiation of macrophages in TB. (A) With the publicly available single-cell data on blood monocytes combined with our macrophages of BALF from LTBI and TB, we embedded on a diffusion map to infer the future fate of macrophages using RNA velocity analysis. (B) UMAP density plots showing distribution of macrophage subtypes identified from PBMC and BALF. The specific markers for identifying type are indicated (right panel). (C) The PAGA connection between two clusters. The weighted edges corresponding to the connectivity between two clusters. The direction of the arrows indicate the transition direction between two clusters. (D) The expression of different cytokines in the supernatant of bronchoalveolar from HC (*n* = 8), LTBI (*n* = 6), TB (*n* = 13), and TBN (*n* = 8). Data were analyzed using a two-sided Student’s t-test (*p < 0.05, **p < 0.01). (E) Schematic plot of experimental design for the human blood-derived macrophages (*n* = 5). Bulk-seq for macrophages after stimulation with or without H37Rv, or cytokines for 24 h. (F) Heatmap indicates the gene expressions comparing different stimulated conditions. (G) Differential pathways enriched in different stimulated conditions by GSVA, and the top 10 enriched gene ontology terms (Biological Process) in distinct comparisons.

We found that the expression of CD14 was high in MM (Figure 2(b)). We hypothesized that the MM may be differentiated from peripheral monocytes. We integrated the public single-cell RNA data of CD14 positive peripheral monocytes [7] and the macrophages in the BALF from LTBI and TB and embedded them on a diffusion map to infer the future fate of macrophages using RNA velocity analysis (Figure 5(a and b)). We identified RNA velocities from CD14 positive monocytes in three different MMs (Figure 5(c)). These data suggested that peripheral monocytes can potentially differentiate into lung macrophage subsets after Mtb infection.

The local lung microenvironment affects macrophage differentiation after Mtb infection [8]. We detected the expression of several cytokines in the BALF supernatant (Figure 5(d)). TB had the highest levels of inflammatory cytokines, particularly IFNγ, IL-6, and TNFα. To define the factors that shape disease-associated macrophage states in affected tissues, we generated human monocyte-derived macrophages, activated with three pro-inflammatory cytokines or H37Rv (Figure 5(e)) and performed RNA-seq analysis. We found that a series of chemokines, such as *CCL23*, *CCL20*, and *CXCL5*, were highly expressed after H37Rv stimulation alone, similar to MM-CCL23 cells defined in our scRNA-seq data (Figure 3(i)). However, the expression of inflammatory chemokines, such as *CXCL9, CXCL10,* and *CXCL11*, was highly upregulated upon cytokines stimulation, especially with IFNγ (Figure 5(f)). GO analysis revealed that the DEGs with upregulated expression were enriched in the chemokine response pathway after H37Rv stimulation (Figure 5(g)), similar to those in LTBI (Figure 3(g)). Nonetheless, the DEGs with upregulated expression were enriched in the IFNγ pathway, the response to molecules of bacterial origin, and the response to lipopolysaccharide in the stimulation of cytokines (Figure 5(g)), similar to the expression of those in TB (Figure 3(g)). These results suggested that Mtb infection and pro-inflammatory cytokines may induce peripheral monocyte differentiation into various MM subsets, which further corroborates the outcome of the disease.

### Interaction between MM cells and CD8 T cells features LTBI

**Figure 6.**
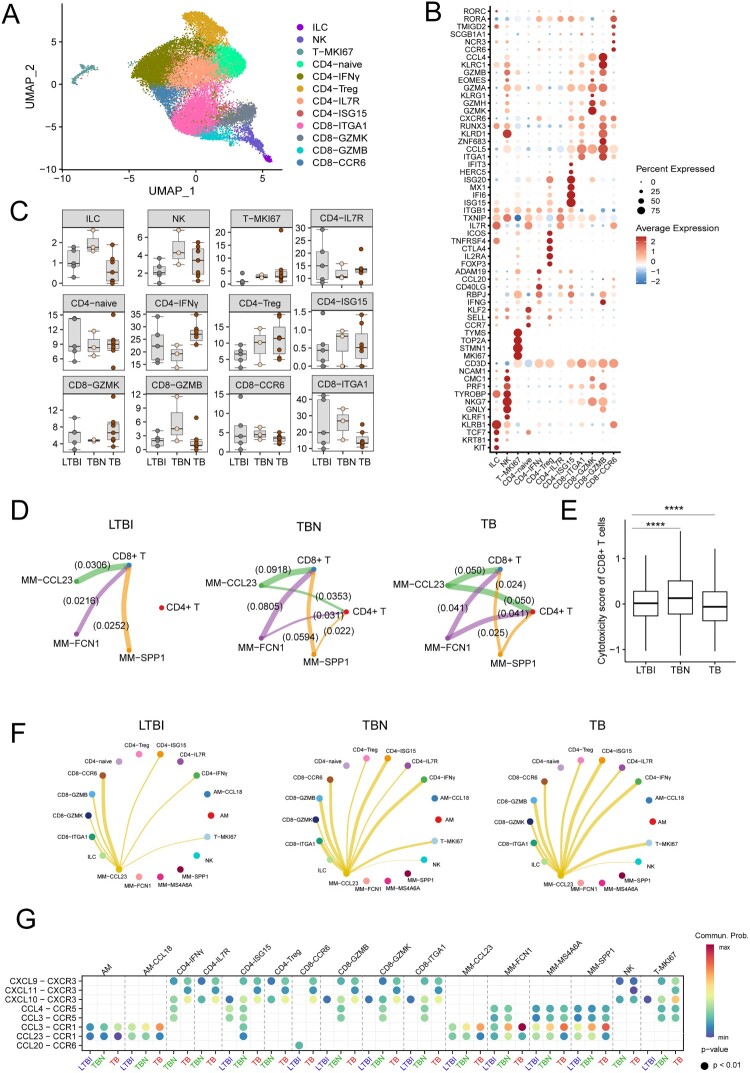
The interaction of T cells and macrophages in BALF cells. (A) UMAP plot of the 10 T-cell types in BALF cells from LTBI (*n* = 5), TB (*n* = 7), and TBN (*n* = 3). (B) Specific markers for identifying each immune cell type. (C) Box plots showing the proportion of each T-cell type in tissues among individual subjects. (D) The interaction of CD4 and CD8 T cells with different macrophage subsets in different individuals. The strength of interaction between cell clusters was indicated. (E) The score of cytotoxicity of CD8 T cells in different individuals. Data were analyzed using a two-sided Student’s t-test. (****p < 0.0001). (F) The interaction of T cells and MM-CCL23 in different individuals. (G) The dot plot of ligand-receptor (L-R) pairs of MM-CCL23 between other subsets of macrophages or different T cells.

We next explored how these macrophage subsets regulate T cells in TB. Re-clustering analysis yielded 12 subpopulations, including 10 clusters of T cells, one NK cell and one innate lymphoid cell (ILC) (Figure 6(a and b), Figure S4A). T cell populations were divided into five CD4 T subpopulations (CD4-naïve T, CD4-IFNγ, CD4-Treg, CD4-ISG15, and CD4-IL7R), four CD8 T subpopulations (CD8-GZMK, CD8-GZMB, CD8-CCR6, and CD8-ITGA1), and proliferating subpopulations (T-MKI67). The proportions of these cell subsets were generally similar. The percentages of CD4-IFNγ, CD4-Treg, CD4-ISG15, and CD8-GZMB were higher in TB than in LTBI and TBN, whereas CD4-naive, CD4-IL7R, CD8-ITGA1, and CD8-CCR6 showed higher percentages in LTBI (Figure 6(c), Figure S4B).

To explore how macrophages regulate these T-cell subsets, we utilized a set of ligand–receptor (L-R) pairs to gain insights into the relationships among cell clusters. We found that the interactions between macrophages and CD8 T cells were prominent in LTBI. However, an additional interaction between macrophages and CD4 T cells was observed in TBN despite being weaker than that in CD8 T cells, which was significantly increased in TB (Figure 6(d)). Interestingly, the cytotoxicity function of CD8 T cells was the highest in LTBI (Figure 6(e)). The interaction between MM-CCL23 and CD8-CCR6 was stronger than that in other subsets in LTBI. However, the interaction between MM-CCL23 and CD4-ISG and CD4-IFNγ was stronger in TBN and TB (Figure 6(f)). CD8-CCR6 exhibited a Tc17-like phenotype, with high expression of *RORA*, *CCR6,* and *KLRB1* in LTBI (Figure S4C). The interaction between MM-CCL23 and CD8-CCR6 was mediated by CCL20 and CCR6 in LTBI (Figure 6(g)). However, the interaction between MM-CCL23 and CD4 and CD8 T cells in TB was mediated by CXCR3 and its ligands CXCL9, CXCL10, and CXCL11 (Figure 6(g), Figure S4D). These cellular interaction analyses revealed axes of cellular plasticity and ligand pleiotropy in different disease stages.

## Discussion

After Mtb infection there are two typical manifestations, LTBI and active TB, representing Mtb control and progression, respectively [9]. Immune parameters that mediate disease control or progression are not clearly defined in humans. In this study, we used scRNA-seq to comprehensively characterize the immune landscape in the BALF at different stages of disease, which provided detailed insights into previously unknown immune protective factors.

Macrophages are highly heterogeneous populations that are strategically located throughout the tissues and organs where they take up and process Mtb [2]. It is widely accepted that macrophage polarization has been classically clustered into two major programmes, classically activated macrophages (M1) and alternatively activated macrophages (M2) [10]. Although previous studies have noted that this paradigm fails to adequately capture the phagocyte heterogeneity observed *in vivo*, the broad implications of this model have persisted, and the functional significance of macrophage heterogeneity has remained underappreciated. Recent studies have provided an in-depth analysis of macrophages stimulated by Mtb using single-cell technology [11, 12]. These results demonstrated the complexity of the macrophage populations in Mtb-infected lungs. In our study, two major subsets of macrophages, AM and MM, were identified. Healthy human alveoli were dominated by AM, which exhibited the function of maintaining homeostasis of the lungs, such as lipid metabolism. However, the proportion of AM was dramatically decreased during TB, possibly due to the increase in other cells, such as T cells infiltrating the lung, or the death of AM induced by tuberculosis infection [13, 14].

Interestingly, three populations of MM cells were identified: MM-FCN1, MM-SPP1, and MM-CCL23. For MM-FCN1, signaling by PRRs (*TLR4* and *TLR2*), could activate specific programmes related to the production of ROS in a Nox2-dependent manner [15]. Moreover, MM-FCN1 exhibited an inflammatory phenotype, including many canonical IFN-I signaling pathways, and responded to IFNγ genes. These IFN-inducible transcripts were significantly highly expressed in the BALF of TB, which was consistent with their high expression in the blood from TB and TB progressors [16, 17], and the IFN-responsive macrophage population in TB-infected macaques [3]. These results indicate that the proportion of inflammatory macrophages increases with the progression of TB infection.

Importantly, MM cells have unique immune characteristics in LTBI, especially for MM-CCL23 cells identified in this study. MM-CCL23 cells were predominantly present in LTBI and associated with cell chemotaxis, as reflected by the unique chemokine profiles *CXCL5*, *CCL20*, and *CCL23*. These chemokines are increased following stimulation with Mtb [3, 18]. However, the expression and function of these chemokines in LTBI remains unclear. Another series of chemokines, including *CXCL9*, *CXCL10*, and *CXCL18*, are highly expressed in TB. Our study and other previous studies used *CXCL10* and *CXCL9* to diagnose active TB [19, 20]. We evaluated the extent to which the immunoregulatory features identified in BALF manifested in the peripheral blood. In a multi-cohort meta-analysis, the expression of *CXCL9* could be a biomarker to distinguish between HC and TB. However, its expression between the LTBI and HC groups was minimal. In contrast, the expression of *CCL23* was higher in LTBI than HC. The chemokine profiles in our study may be diagnostic biomarker candidates for tuberculosis.

T cells are critical for the host immune response to Mtb infection. It is widely accepted that CD4 T cells are critical for resistance to Mtb [21, 22], whereas CD8 T cells were initially thought to be less important than CD4 T cells [22, 23]. CD8 T cells may play a critical but complex role in TB infection [24]. Several lines of evidence show the importance of CD8 T cells in controlling initial or long-term Mtb infection. Andersen et al. suggested that CD8 T cells were not involved in the acute stage of TB infection, but this subset was active and produced IFNγ during the latent phase of infection [25]. Interestingly, we found that CD8 T cells directly interacted with macrophages more strongly than CD4 T cells in LTBI. Moreover, the cytotoxicity of CD8 T cells was the highest in LTBI. These data suggested that cytotoxic-CD8 T cells may play an important role against Mtb in LTBI. Evidence supporting this notion showed that IFN- and ISG-related gene expression in macrophages and T cells was not high in LTBI. Furthermore, the interaction between CD8-CCR6 and MM-CCL23 cells was the strongest in LTBI. CD8-CCR6 cells exhibited a Tc17-like phenotype, with high expression of *RORC, RORA*, and *KLRB1*. Tc17 was found in tuberculous pleural effusions from TB and CCR6 expression [26]. However, the role of Tc17 in TB remains unclear*.* These results indicated that the immune role of CD8 T cells may be important in the early stages of tuberculosis, which may inform future vaccine design for early protective immunity.

Furthermore, we found that the profile of immune cells in TBN was different from that in LTBI and TB. For example, the pathways of IFNγ, response to lipopolysaccharide, and response to TNFɑ in MM-FCN1 cells were higher in the TBN than in the LTBI but lower than TB. However, the expression of chemokines (such as *CCL20*, *CCL23* and *CXCL5*) in MM-CCL23 was lower in the TBN than LTBI but higher than TB group. Of particular interest was the interaction of macrophages with T cells, which was unique in the TBN compared to that LTBI and TB. Therefore, we speculated that the immune profile of the TBN might be in a “standby” or “early disease” state, which may assist in the development of future preventive treatments for tuberculosis.

Altogether, this comprehensive cell census revealed that distinct types of macrophages in BALF were associated with TB disease status, and MM-CCL23 was predominantly present in LTBI. These MM-CCL23 cells possibly function against TB by interacting with CD8 T cells. However, the functions of LTBI-specific macrophages and T cells need to be further verified.

In conclusion, using our generalization framework, we identified the immune landscape of human BALF from different stages after Mtb infection, which has important implications for understanding the immunological basis of TB control through the development of host-directed immunotherapies or vaccines.

## Supplementary Material

Supplemental Material
