# Supplementary figures and images for: The interaction of macrophages and CD8 T cells in bronchoalveolar lavage fluid is associated with latent tuberculosis infection
Source: Emerg Microbes Infect. 2023 Aug 2;12(2):2239940. doi: 10.1080/22221751.2023.2239940 (PMC10399483; doi:10.1080/22221751.2023.2239940)

Figure S1

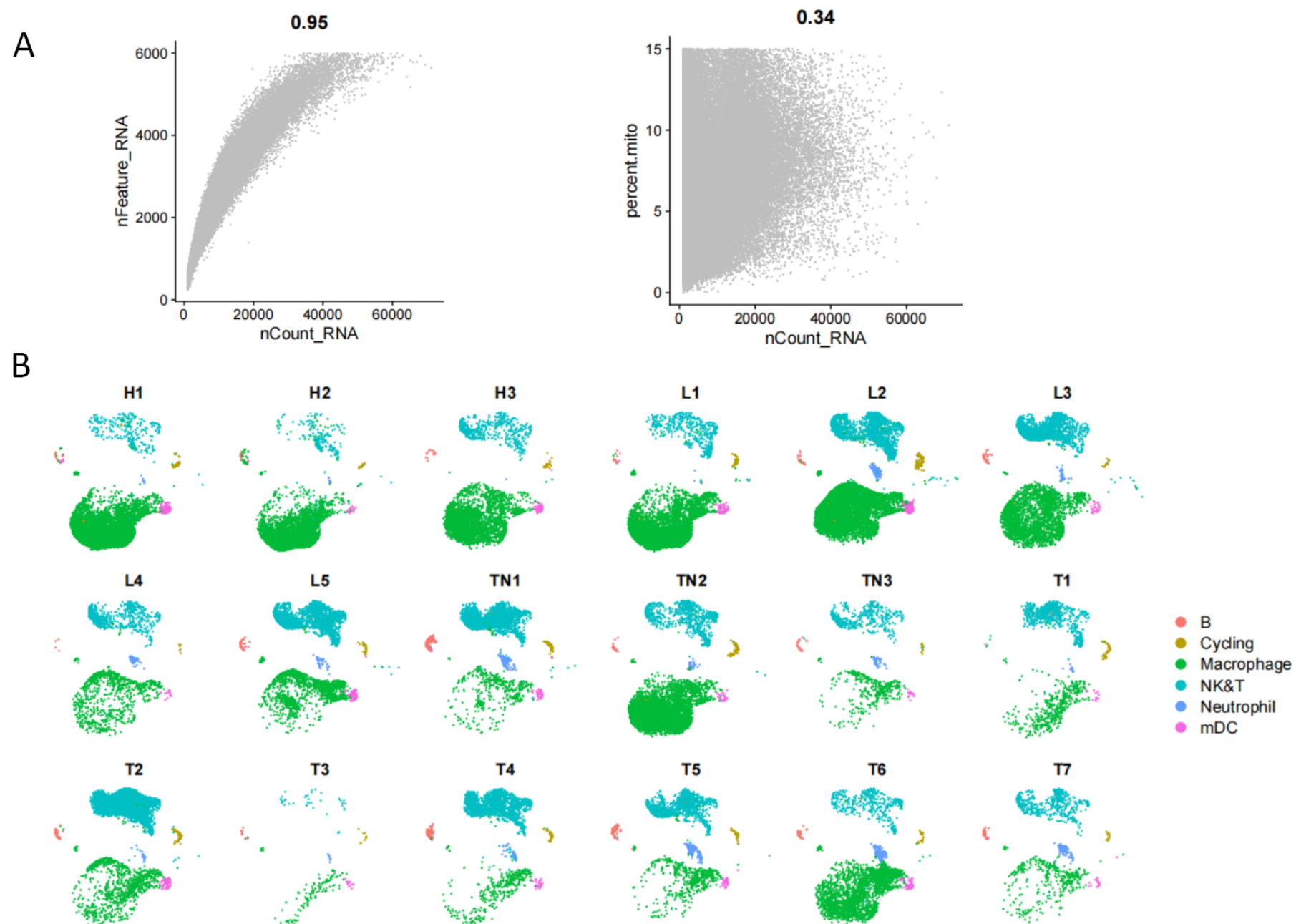

Figure S2

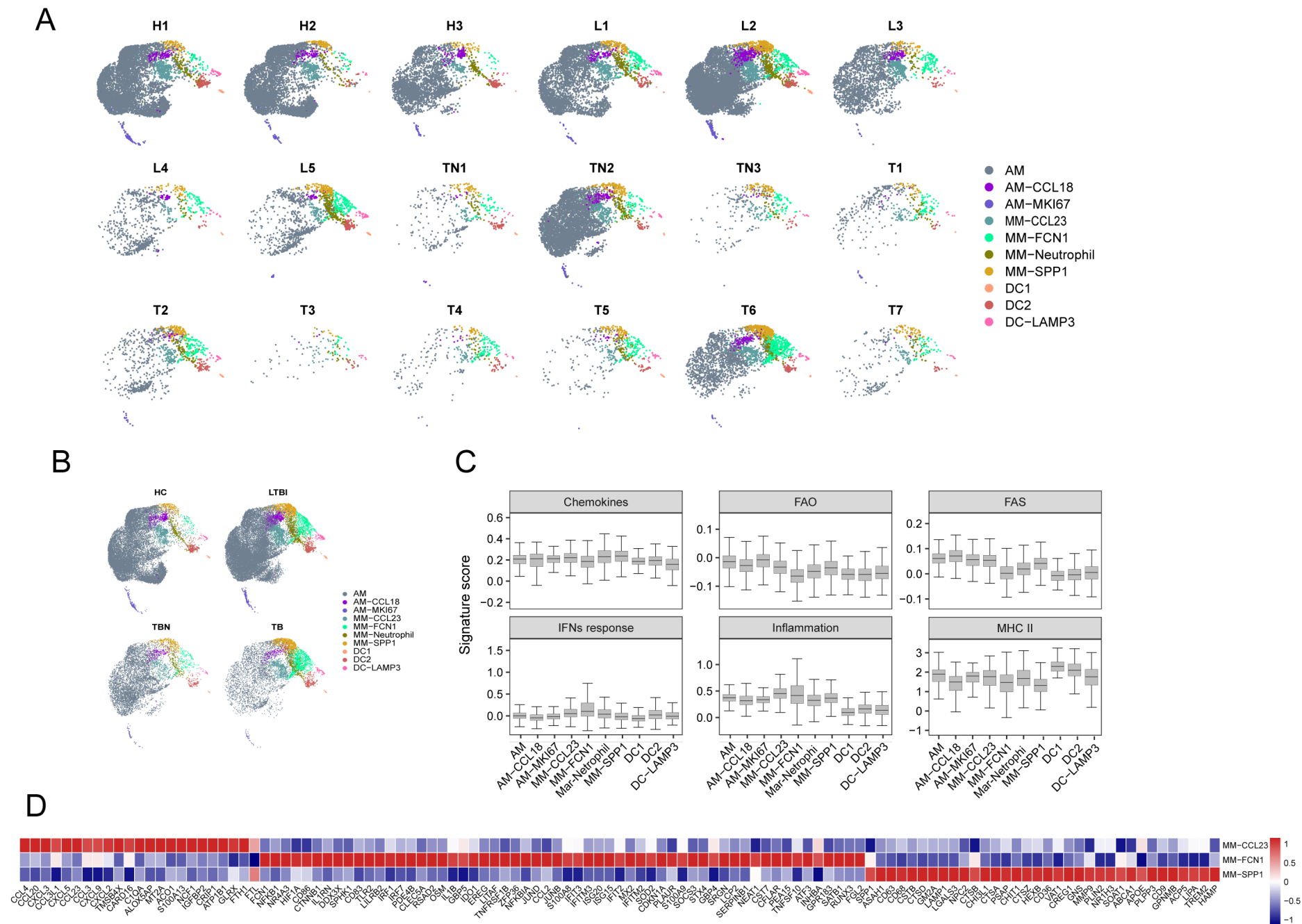

Figure S3

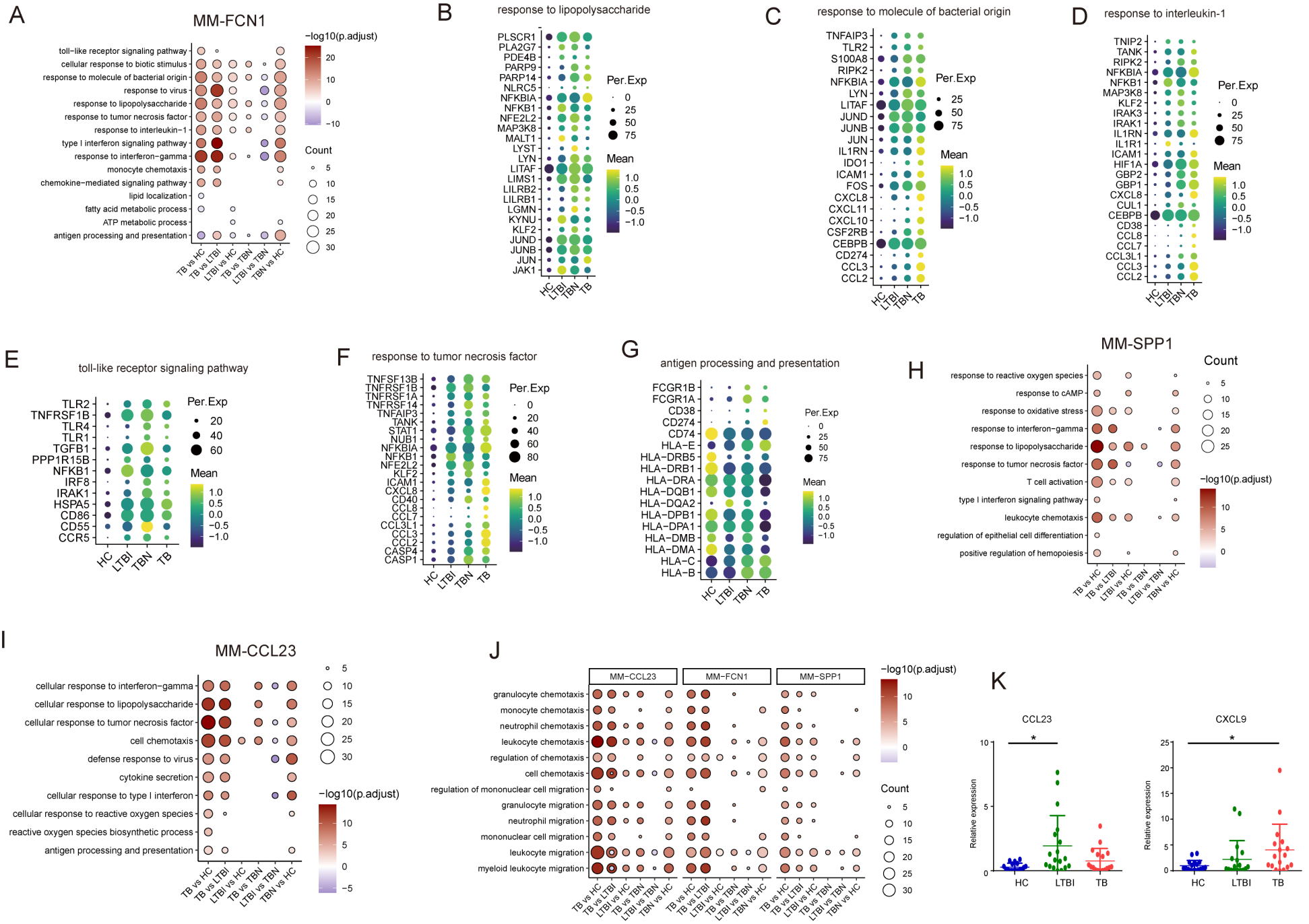

Figure S4

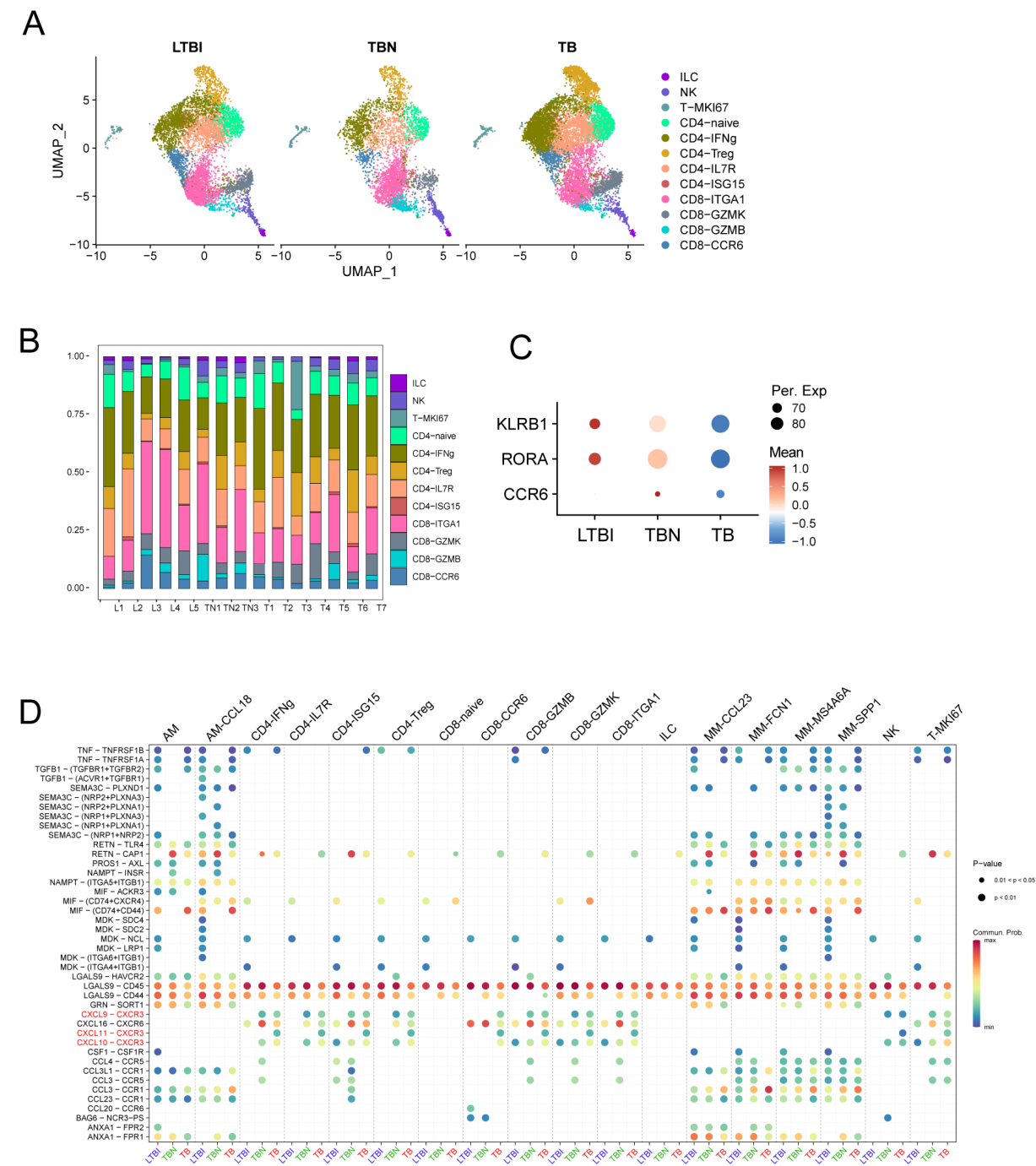

Supplement: Supplemental Material [file TEMI_A_2239940_SM7503.zip › supplymentary Figures.pdf]
